# Obstetric fistula repair failure and its associated factors among women underwent repair in Yirgalem Hamlin fistula center, Sidama Regional State, Southern Ethiopia, 2021: a retrospective cross sectional study

**DOI:** 10.1186/s12905-022-01866-z

**Published:** 2022-07-10

**Authors:** Shimelis Tadesse, Neway Ejigu, Dejene Edosa, Tebeje Ashegu, Dubale Dulla

**Affiliations:** 1 Department of Midwifery, College of Health Sciences, Mettu University, P.O. Box: 318, Mettu, Ethiopia; 2 Department of Midwifery, College of Medicine and Health Sciences, Madda Walabu University, P.O. Box: 247, Bale Robe, Ethiopia; 3 Department of Midwifery, College of Medicine and Health Sciences, Salale University, P.O. Box: 245, Fiche, Ethiopia; 4 Department of Midwifery, College of Medicine and Health Sciences, Hawassa University, P.O. Box: 1560, Hawassa, Ethiopia

**Keywords:** Obstetric fistula repair failure, Fistula center, Southern Ethiopia

## Abstract

**Background:**

Obstetric fistula repair failure is a combination of unsuccessful fistula closure and/or incontinence following a successful closure. It causes a burden on both the patients and the fistula centers. The aim of this study was to assess the magnitude and associated factors of obstetric fistula repair failure among women who underwent fistula repair at Yirgalem Hamlin fistula center in Southern Ethiopia.

**Methods:**

A facility-based retrospective cross-sectional study was conducted among women who underwent fistula repair at Yirgalem Hamlin fistula center, Southern Ethiopia, during the period from January 2016 to December 2020. All 562 women who underwent fistula repair in the last 5 years were included in the study. The data were collected using a pre-tested checklist from September 22 to October 22, 2021. The data were then imported into EPI info-data version 3.1, exported to SPSS version 25, and analyzed. Descriptive and logistic regression analyses were performed, and the significant statistical test was assessed at a 95% confidence interval. Variables with a *p* value of < 0.05 in multivariable logistic regression were regarded to have a statistically significant relationship.

**Results:**

The magnitude of obstetric fistula repair failure in this study was 28.8%. Obstetric fistula repair failure was found to be associated with labor duration > 48 h (AOR = 2.037; 95% CI 1.268, 3.272), Goh Type 4 fistulas (AOR = 3.939; 95% CI 1.623, 9.560), fistula size > 3 cm (AOR = 6.627; 95% CI 3.802, 11.554), completely destructed urethra (AOR = 3.192; 95% CI 1.234, 8.256), and bladder catheterization > 14 days (AOR = 2.944; 95% CI 1.380, 6.281).

**Conclusions:**

The magnitude of obstetric fistula repair failure was significantly higher than the World Health Organization standard. Obstetric fistula repair failure had a positive association with a longer duration of labor, Goh Type 4 fistulas, large fistula size, total urethral injury, and a longer period of bladder catheterization. Therefore, the concerned bodies need to implement interventions on factors affecting obstetric fistula repair failure to reduce or prevent the failure of obstetric fistula repair.

**Supplementary Information:**

The online version contains supplementary material available at 10.1186/s12905-022-01866-z.

## Introduction

Obstetric fistula is defined as an abnormal opening that occurs because of prolonged pressure from a fetal head on the mother’s pelvic bones (in the case of obstructed labor) that damages the genital organs and surrounding structures. It mostly involved the bladder and the vagina (vesicovaginal fistula-VVF), followed by the rectum and the vagina (rectovaginal fistula-RVF). Even though different types of fistula can occur between the genital and surrounding organs, VVF and RVF are the most common. This disorder leads to victims having continuous urine or feces leaking through their vagina [1, 2].

Other than the involvement of the bladder to other genital organs, obstetric fistula can be further classified based on anatomical locations, such as urethrovaginal fistula (between the urethra and the vagina), urethrocervical fistula (between the urethra and the cervix), ureterovaginal fistula (between the ureters and the vagina), and vesicouterine fistula (between the bladder and the uterus) [1, 3].

Obstetric fistula repair is critical to a woman’s overall well-being or quality of life [4]. Accurate diagnosis, pre-operative care, prompt repair, employing basic principles of surgical procedures with or without interposition flaps, post-operative care, and follow-up are all required for obstetric fistula repair [3, 4].

There are three possible outcomes for women who undergo obstetric fistula repair. These are closed fistula and continents (or closed and dry), failed fistula closure, and incontinence after successful fistula closure. As a result, closed fistula and continence are considered successful repair outcomes [7, 8]. On the other hand, obstetric fistula repair failure was indicated by the combination of repair outcomes of failed fistula closure and incontinence following successful closure [7–9].

The World Health Organization (WHO) set a goal of less than 15% for failed fistula closure after repair and less than 10% for incontinence after successful fistula closure as the ideal range of repair outcomes to determine the level of quality of services given to patients [12]. On the other hand, the magnitude of obstetric fistula repair failure is context-dependent [13]. In Pakistan (South Asia), for example, the magnitude of obstetric fistula repair failure was reported as 12.8% [8]. In African countries, for instance, it ranges from 11% in Uganda [14] to 58% in Angola [7].

In Ethiopia’s Bahir Dar Hamlin fistula center, 35.3% of patients had an obstetric fistula repair failure [15]. Furthermore, a prior study conducted at Yirgalem Hamlin fistula center revealed that 18.3% of fistulas failed to close. However, incontinence was not studied following successful closure [15]. Obstetric fistula repair failure was found in 15.5% of patients at Ethiopia’s Jimma University teaching hospital [16].

Socio-demographic factors such as age at the time of repairs [17] and weight less than or equal to 50 kg [18], as well as obstetric factors such as home delivery [18], vaginal delivery [10], and labor duration of 2 days or more [16, 17] were found to be risk factors for fistula repair failure in various studies. Fistula characteristics such as large fistula size [6, 16, 18, 19], Goh Type 3, or Type 4 fistulas [16, 18], past repair history [6, 17, 18], urethral damage [8, 19], and moderate to severe vaginal scarring [18, 19] have also been described as risk factors for repair failure. Peri-operative factors such as surgeons’ experience [22], abdominal repair [16], bladder catheterization time [23], and postoperative infection [18] were also examined as contributing factors to obstetric fistula repair failure.

Due to the unfavorable outcomes, patients are suffering physical, psychological, and social problems [24]. As a result, obstetric fistula repair failure imposes an additional burden on patients, as well as on treating institutions or fistula centers [25]. It leads to repeated surgery and as a result, there is a higher risk of failed fistula closure in repeated repairs [26] because the first surgical repair is one of the factors for successful closure of the fistula [6]. The average cost for obstetric fistula patient treatments including repair, postoperative care, and rehabilitation services is estimated at 450 US dollars [27].

Hence, improving the nutritional status of women, having trained surgeons, building adequate facilities that give operation services, giving education to patients, motivating surgeons through increasing salaries, and addressing the transportation problems of patients are among the possible solutions to overcome obstetric fistula repair failure [3, 24]. On the whole, availability, and quality of emergency obstetric and newborn care, community awareness, involvement, and strong political commitment toward effective fistula care are the cornerstones for preventing obstetric fistula repair failure [17, 25].

The awareness about the magnitude and risk factors of failure of fistula repair for women who undergo fistula repair may help to increase the quality of care of patients among health care providers working in fistula centers and improve the overall outcome of fistula repair in Ethiopia. However, the magnitude and associated risk factors of fistula repair failure among women who underwent fistula repair have not yet been sufficiently investigated in our study area. Therefore, the purpose of this study is to evaluate obstetric fistula repair failure and its associated factors in women who underwent fistula repair at Yirgalem Hamlin fistula center for the last 5 years.

## Methods

### Study design, period, and setting

A facility-based retrospective cross-sectional study was conducted at Yirgalem Hamlin fistula center, Southern Ethiopia for five years (from January 2016 to December 2020), and data were collected from September 22, 2021, to October 22, 2021. Yirgalem Hamlin Fistula center is one of Hamlin Fistula’s centers founded by Dr. Catherine Hamlin and Dr. Reginald and established in 2006. It is found in the Sidama Regional State and is situated 300 km away from the capital city, Addis Ababa, Ethiopia. Annually, it ensures over 400 surgeries for different cases, such as urogenital fistula, pelvic organ prolapse, and uterine vaginal prolapse. So far, 2578 fistula surgeries have been performed since its establishment. However, in the last 5 years from January 1, 2016, to December 31, 2020, about 610 women underwent fistula repair. The center provides services with one medical doctor (senior gynecologist), 1 Midwife, 4 Health officers, and 14 trained aid nurses; and has 38 beds.

### Population and eligibility criteria

Women who underwent obstetric fistula repair at Yirgalem Hamlin fistula center between January 1, 2016, and December 31, 2020, were included in this study.

Medical records of all the study population were reviewed in the study. However, those with incomplete data records, those who missed the postoperative care follow-up, and fistulas caused by non-obstetric causes (such as hysterectomy or rape) were excluded from the study.

### Sample size, sampling techniques, and procedures

The study included all women who underwent obstetric fistula repair in the last 5 years, from January 1, 2016, to December 31, 2020 (n = 562). All medical charts of women (using the census method) who underwent fistula repair and met the eligibility criteria from January 1, 2016, to December 31, 2020, were included in the study. The card numbers were identified from the registration book of women who underwent obstetric fistula repair and were registered between January 2016 and December 2020. Accordingly, 610 cards were identified as having had a fistula repair in the previous 5 years. All of the cards were reviewed to ensure that they had all of the information required for the study.

**Fig. 1:**
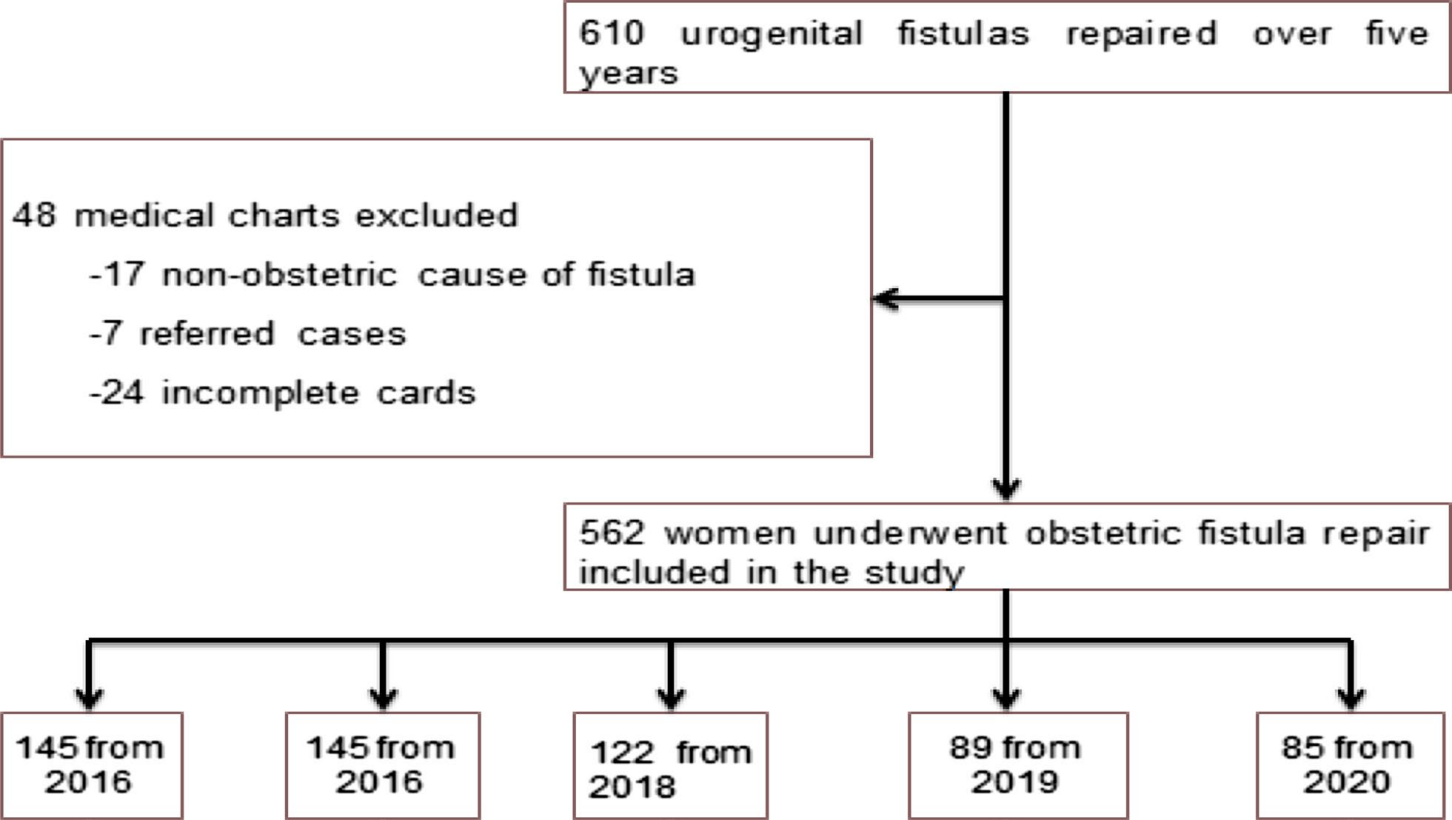
Flow diagram for women underwent fistula repair included for the study in Yirgalem Hamlin fistula center, Southern Ethiopia, 2021

Mothers' cards that met the inclusion criteria were 562, but 24 cards were incomplete, 17 cards were the non-obstetric causes of fistula, and 7 of them were referred cases and excluded from the study (Fig. 1).

### Data collection tools and procedures

The data collection checklist was adapted from the national fistula patient management-recording format and different literature related to the study title. The information was gathered from the patient's chart, operation logbook, and discharge logbook over a five-year period (January 1, 2016, to December 31, 2020). Four data collectors and one supervisor with a background of one Nurse and four Midwives who have a Bachelor of Science degree were assigned. Using a pretested data collection checklist, data on sociodemographic characteristics, obstetric characteristics including comorbidities, fistula characteristics, perioperative characteristics, and obstetric fistula repair failure were collected.

### Validity and reliability of data collection checklist

The validity and reliability were assured by using the national fistula patient management recording format and previous related studies. In addition, a pretest was carried out, and experts have reviewed the data collection checklist. Moreover, the tool reliability test was checked by Cronbach's alpha.

### Data processing and analysis

After the data collections were completed, data were checked for completeness, coded, and cleaned. After entering the data into EPI-info data version 3.1, it was exported to IBM SPSS version 25.0 (IBM Corporation, Armonk, NY, UAS) for analysis.

Descriptive statistics were used to describe the various variables using a table, pie chart, bar graph, and line graph. Inferential statistics of binary logistic regression analysis was performed to examine the relationship between the various factors and the outcome variable. In bivariate logistic regression, variables having a p-value less than 0.20 were put into multivariable logistic regression. For statistical significance tests, frequency distributions and percentages with a 95% confidence level were calculated. In multivariable logistic regression, variables with a p-value less than 0.05 were regarded to have a statistically significant relationship with the outcome variable.

### Study outcomes

The main outcomes of this study were the magnitude of obstetric fistula repair failure and associated factors.

### Operational definition

Obstetric fistula repair failure is the status of failed fistula closure and/or incontinence after successful fistula closure 21 days after surgery had been done.

Goh Type fistula classification is the classification system that measures the distance of the distal edge of the fistula from the external urethral meatus (for VVF) or the hymen (for RVF). Based on this, Type 1 fistula is > 3.5 cm, Type 2 fistula is between 2.5 and 3.5 cm, Type 3 fistula is between 1.5 and 2.5 cm, and Type 4 fistula is < 1.5 cm from the external urethral meatus or hymen (see Additional file 1) [29]. 

Number of fistula is the number of abnormal holes formed among organs (between either the bladder and the vagina or the rectum and the vagina) due to genitourinary fistula occurrence, which might be single or multiple.

Circumferential defect is a condition of the separation of the urethra from the bladder due to fistula formation [30].

### Data quality control

To assure the quality of data, two days of training were given to data collectors and a supervisor before data collection on the objective of the study, the use of institution recording forms, as well as the importance of filling in the data collection checklist completely and accurately. In addition, a pretest was carried out on 35 (5% of total reviewed women) medical records of women who underwent fistula repair in 2015 and 2021 at Yirgalem Hamlin fistula center to check the appropriateness of the data collection checklist, to collect information, and to minimize or avoid missing information. Moreover, a code was given for each questionnaire, close supervision was employed by the supervisor, and daily activities were checked every day after data collection for completeness, clarity, and consistency by the supervisor and principal investigator.

## Results

### Sociodemographic characteristics

Five hundred sixty-two (562) complete cases were included in the study. The mean age at the time of repair was 30.7 (SD ± 10.0), with a range from 14 to 61 years. Nearly half of the patients, 273 (48.6%), were between 20 and 34 years old. The majority, 353 (62.8%), have no formal education and 179 (31.9%) have some formal education. Nearly three in four (71.2%) of the study participants weighed below 50 kg and the mean was 47.3 (SD ± 7.3), while 336 (59.8%) women had a height above 150 cm and the mean was 152.2 (SD ± 7.3) (Table 1).

**Table 1:** Sociodemographic characteristics of women underwent obstetric fistula repair in Yirgalem Hamlin fistula center, Southern Ethiopia, 2021

Variables	Category	Frequency (n = 562)	Percent (%)
Age at repair in years	< 20	96	17.1
	20–34	273	48.6
	> 34	193	34.3
Educational status	Have no formal education	353	62.8
	Have some formal education	179	31.9
	Unknown^a^	30	5.3
Weight in kilograms	< 50	400	71.2
	≥ 50	162	28.8
Height in centimeters	< 150	226	40.2
	≥ 150	336	59.8

^a^Represent data not recorded

### Obstetric characteristics including comorbidities

The mean age of study subjects at first pregnancy was 17.9 (SD ± 2.5), with a range from 12 to 28. The majority of the 440 (78.3%) were stillbirths, and more than half of the patients, 305 (54.3%), did not have antenatal care (ANC) follow-up for their causative delivery. Regarding the place of delivery, almost a quarter, 144 (25.6%), were delivered at home, and 194 (34.5%) had greater than 48 h of labor duration (includes all stages of labor). Furthermore, about 8 (1.4%) of repairing women had comorbidities (2 (0.4%) had HIV and 6 (1.0%) had foot drops) (Table 2).

**Table 2:** Obstetric fistula characteristics of women who underwent obstetric fistula repair in Yirgalem Hamlin fistula center, Southern Ethiopia, 2021

Variables	Category	Frequency (n = 562)	Percentage (%)
Age at first pregnancy	< 18	375	66.7
	≥ 18	187	33.3
Number of pregnancies (gravida)	I	193	34.3
	II-IV	205	36.5
	≥ V	164	29.2
Number of delivery (parity)	One	207	36.8
	More than one	355	63.2
Number of alive children	None^a^	132	23.5
	1–4	326	58.0
	≥ 5	104	18.5
Presence of ANC	No	240	42.7
	Yes	305	54.3
	Unknown^b^	17	3.0
Place of delivery	Home	144	25.6
	Health center	131	23.3
	Hospital	287	51.1
The total duration of labor in hours	≤ 48	368	65.5
	> 48	194	34.5
Mode of delivery	Spontaneous vaginal delivery	306	54.4
	Instrumental vaginal delivery	28	5.0
	Cesarean section	228	40.6
Feta outcome	Stillbirth	440	78.3
	Live birth	122	21.7
Comorbidities (HIV and/or foot drops)	No	554	98.6
	Yes	8^c^	1.4

^a^Women who had not alive children due to child (children died); ^b^data not recorded while ^c^represents comorbidities of those two women had HIV and six women had foot drops

### Fistula characteristics

**Fig. 2:**
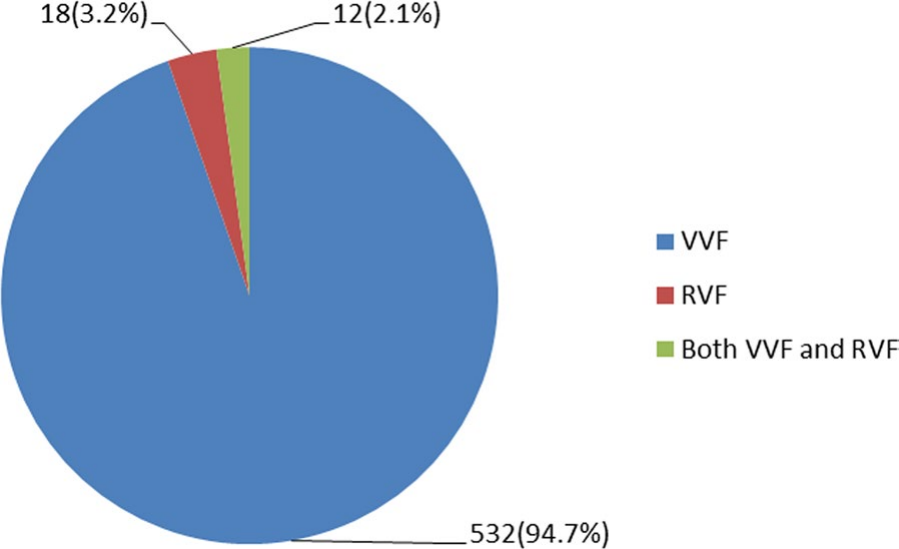
Fistula type of women underwent obstetric fistula repair in Yirgalem Hamlin fistula center, Southern Ethiopia, 2021

Approximately one-fifth, 108 (19.2%) of study participants had been living with an obstetric fistula for ≥ 60 months. Regarding the Goh Type of classification, the majority, 235 (41.8%) of women who participated in this study had type two fistulas. Furthermore, nearly one-fifth of the 107 (19.0%) had a large fistula size of > 3 cm (Table 3). The majority, 532 (94.7%) of study participants’ cases were VVF and 18 (3.2%) were RVF, while the rest of the cases were both VVF and RVF (Fig. 2).

**Table 3:** Fistula characteristics of women who underwent obstetric fistula repair in Yirgalem Hamlin fistula center, Southern Ethiopia, 2021

Variables	Category	Frequency (n = 562)	Percentage (%)
The total duration of a fistula to repair in months	≤ 12	364	64.8
	12–60	90	16.0
	≥ 60	108	19.2
Goh fistula type	Type 1	177	31.5
	Type 2	235	41.8
	Type 3	106	18.9
	Type 4	44	7.8
Fistula size in centimeters	≤ 3	455	81.0
	> 3	107	19.0
Presence of circumferential defect	No	468	83.3
	Yes	94	16.7
Previous history of fistula repair	No	553	98.4
	Yes	9	1.6
Number of fistulae	One	534	95.0
	Two	28	5.0
Status of urethra	Intact	316	56.2
	Partially damage	208	37.0
	Completely damage	38	6.8
Status of the bladder neck	Intact	450	80.1
	Damage^a^	112	19.9
Vaginal scarring/fistula fibrosis	None	40	7.1
	Mild	400	71.2
	Moderate to severe	122	21.7

^a^The damage includes partially and completely destructed bladder neck

### Perioperative history

The majority, 553 (98.4%) of obstetric fistula patients’ surgical repairs were done by a senior gynecologist, and all the surgery was done using spinal anesthesia. Almost all surgeries, 561 (99.8%), were done through the transvaginal route of repair. Five hundred thirty-four (95.0%) of the surgeries were done in a primary attempt and more than half, 324 (57.7%) of the surgical repairs were done by one-layer closure. The majority, 508 (90.4%), of women were catheterized for ≤ 14 days and only 24 (4.3%) developed infection after repair (Table 4).

**Table 4:** Perioperative history of women who underwent obstetric fistula repair in Yirgalem Hamlin fistula center, Southern Ethiopia, 2021

Variables	Category	Frequency (n = 562)	Percentage (%)
Surgery is done by	Senior gynecologist	553	98.4
	Guest/resident gynecologist	9	1.6
Surgical approach	Vaginal	561	99.8
	Abdominal	1	0.2
Surgical attempt	Primary	534	95.0
	Second	28	5.0
Layers of closure	One	324	57.7
	Two	238	42.3
Infection after repair	No	538	95.7
	Yes	24	4.3
Number of days to catheter removal	≤ 14	508	90.4
	> 14	54	9.6

### The magnitude of obstetric fistula repair failure

**Fig. 3:**
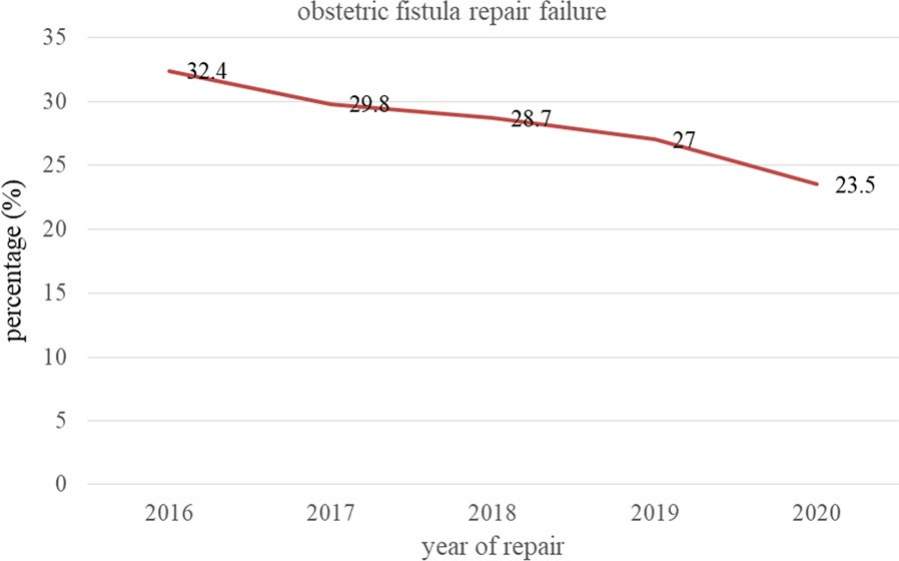
Trend of obstetric fistula repair failure over five years in Yirgalem Hamlin fistula center, Southern Ethiopia, 2021

The magnitude of obstetric fistula repair failure among women who underwent repair was 162 (28.8%). Among the total women who underwent repair (n = 562), 40 (7.1%) had failed fistula closure and 122 (23.4%) were incontinent. Incontinence after successful fistula closure accounts for three-fourths (75.3%) of obstetric fistula repair failure (Table 5). Over the five years, obstetric fistula repair failure decreased from 32.4% in 2016 to 23.5% in 2020 (Fig. 3).

**Table 5:** Magnitude of obstetric fistula repair failure among women underwent fistula repair in Yirgalem Hamlin fistula center, Southern Ethiopia, 2021

Variables	Category	Frequency	Percentage (%)
Failure of fistula closure after 21 days (n = 562)	No	522	92.9
	Yes	40	7.1
Incontinent after successful closure after 21 days (n = 522)	No	400	76.6
	Yes	122	23.4
Obstetric fistula repair failure after 21 days (n = 562)	No	400	71.2
	Yes	162	28.8
Obstetric fistula repair failure (n = 162)	Unclosed	40	24.7
	Incontinent	122	75.3

### Factors associated with obstetric fistula repair failure

In bivariable logistic regression, 14 variables had a statistically significant association with obstetric fistula repair failure at a *p* value < 0.2. These were age at repair, number of deliveries (parity), duration of labor, fetal outcome, fistula type, circumferential defect, Goh fistula Type, fistula size, number of fistulas, the status of the urethra, the status of the bladder neck, level of vaginal scarring, the status of the surgeon, and the number of days of catheter removal.

Women who had > 48 h of labor duration were 2 times more likely to have obstetric fistula repair failure compared to women who had ≤ 48 h of labor duration (AOR = 2.037; 95% CI 1.268, 3.272). Women who had Goh type 4 fistula were 3.9 times more likely for obstetric fistula repair failure compared to women who had Goh type 1 fistula (AOR = 3.939; 95% CI 1.623, 9.560). Furthermore, women who had a large fistula size (> 3 cm) were 6.6 times more likely to have obstetric fistula repair failure compared to women who had a fistula size ≤ 3 cm (AOR = 6.627; 95% CI 3.802, 11.554). Additionally, women with completely destroyed urethras were 3.2 times more likely than women with intact urethras to have obstetric fistula repair failure (AOR = 3.192; 95% CI 1.234, 8.256). Lastly, women who had bladder catheterization during the postoperative time for > 14 days were 2.9 times more likely to have obstetric fistula repair failure compared to women who had bladder catheterization for ≤ 14 days (AOR = 2.944; 95%CI: 1.380, 6.281) (Table 6).

**Table 6:** Binary logistic regression analyses for factors associated with obstetric fistula repair failure among women underwent repair in Yirgalem Hamlin fistula center, Southern Ethiopia, 2021

Variables	Obstetric fistula repair failure N (%)		COR with 95% CI	AOR with 95% CI	*p* -value
	No	Yes			
Age at repair					
< 20	62 (64.6)	34 (35.4)	1	1	
20–34	210 (76.9)	63 (23.1)	0.547 (0.330, 0.906)	0.705 (0.351, 1.417)	0.326
> 34	128 (66.3))	65 (33.7))	0.926 (0.554, 1.548)	1.555 (1.043, 5.859)	0.277
Number of delivery (parity)					
One	131 (63.3)	76 (36.7)	1	1	
More than one	269 (75.8)	86 (24.2)	0.551 (0.380, 0.800)	0.554 (0.305, 1.005)	0.052
Duration of labor					
≤ 48	290 (78.8)	78 (21.2)	1	1	
> 48	110 (56.7)	84 (43.3)	2.839 (1.945, 4.145)	2.037 (1.268, 3.272)	0.003**
Fetal outcome					
Stillbirth	302 (68.6)	138 (31.4)	1.866 (1.143, 3.045)	1.255 (0.680, 2.316)	0.467
Live birth	98 (80.3)	24 (19.7)	1	1	
Fistula type					
VVF	383 (72.0)	149 (28.0)	1	1	
RVF	13 (72.2)	5 (27.8)	0.989 (0.346, 2.821)	1.158 (0.327, 4.098)	0.820
Both VVF and RVF	4 (33.3)	8 (66.7)	5.141 (1.525, 17.327)	1.612 (0.276, 9.428)	0.596
Goh fistula type					
Type 1	141 (79.7)	36 (20.3)	1	1	
Type 2	185 (78.7)	50 (21.3)	1.059 (0.654, 1.713)	0.784 (0.434, 1.405)	0.413
Type 3	56 (52.78	50 (47.2)	3.497 (2.061, 5.933)	1.555 (0.785, 3.079)	0.206
Type 4	18 (40.9)	26 (59.1)	5.657 (2.799, 11.434)	3.939 (1.623, 9.560)	0.002**
Fistula size in cm					
≤ 3	366 (80.4)	89 (19.6)	1	1	
> 3	34 (31.8)	73 (68.2)	8.829 (5.528, 14.103)	6.627 (3.802, 11.554)	0.000***
Circumferential defect					
No	366 (78.2)	102 (21.8)	1	1	
Yes	34 (36.2)	60 (63.8)	6.332 (3.940, 10.177)	1.824 (0.955, 3.484)	0.069
Number of fistulae					
One	389 (72.8)	145 (27.2)	1	1	
Two	11 (39.3)	17 (60.7)	4.146 (1.897, 9.063)	2.639 (0.874, 7.971)	0.085
Status of urethra					
Intact	256 (81.0)	60 19.0)	1	1	
Partially damage	130 (62.5)	78 (37.5)	2.560 (1.721, 3.809)	1.464 (0.845, 2.536)	0.174
Completely damage	14 (36.8)	24 (63.2)	7.314 (3.572, 14.975)	3.192 (1.234, 8.256)	0.017*
Status of bladder neck					
Intact	342 (76.0)	108 (24.0)	1	1	
Damage	58 (51.8)	54 (48.2)	2.948 (1.920, 4.528)	1.252 (0.669, 2.345)	0.482
Level of vaginal scarring/fibrosis					
None	34 (85.0)	6 (15.0)	1	1	
Mild	299 (74.8)	101 (25.3)	1.914 (0.781, 4.693)	1.958 (0.661, 5.798)	0.225
Moderate to severe	67 (54.9)	55 (45.1)	4.652 (1.820, 11.888)	2.020 (0.622, 6.562)	0.242
Surgeon status					
Gynecologist	396 (71.6)	157 (28.4)	1	1	
Guest/Resident	4 (44.4)	5 (55.6)	3.153 (0.836, 11.894)	0.800 (0.121, 5.282)	0.817
Number of days to catheter removal					
≤ 14	378 (74.4)	130 (25.6)	1	1	
> 14	22 (40.7)	32 (59.3)	4.229 (2.372, 7.541)	2.944 (1.380, 6.281)	0.005**

Keys: COR = crude odds ratio; AOR = adjusted odds ratio; 1 = reference category; *p* value significant at: *p < 0.05, **p < 0.01, ***p < 0.001, N = number while (%) represents percentages

## Discussion

In this study, the magnitude and associated factors of obstetric fistula repair failure were determined among women who underwent repair at Yirgalem Hamlin fistula center. According to the result, the magnitude of obstetric fistula repair failure was found to be 28.8% (*p* = 28.8%, 95% CI 23.5, 32.4) and longer duration of labor, Goh Type 4 fistulas, large fistula size, completely destructed urethra, and longer duration of bladder catheterization were significantly associated with obstetric fistula repair failure.

This study showed that the overall magnitude of obstetric fistula repair failure was 28.8% over the five-year period. Among these, 7.1% of women had failed fistula closure, and 23.4% of women were incontinent after successful fistula closure.

The magnitude of obstetric fistula repair failure in this study was consistent with that of studies conducted in Benin (26.8%) [31] and the Democratic Republic of Congo (28.3%) [32]. The findings' consistency could be attributable to the patients' fistula characteristics being similar and comparable data duration being included in the study. Previous research in Benin used five years of data from 2009 to 2013, while data from 2007 to 2013 was used in the Democratic Republic of Congo, and this study included five years of data from 2016 to 2020.

However, the magnitude obstetric fistula repair failure of this study was higher than those of studies conducted in Uganda (11%) [14], Rwanda (14%) [19], the Democratic Republic of Congo (17.1%) [13], Guinea (17.5%) [10], Kenya (18%) [28], and Jimma (Ethiopia) (15.5%) [16]. The possible reasons might be due to the setting differences [33], the difference in the quality of service given to the patients in different areas [23], and the small sample size and inclusion criteria. For instance, in the previous study of Jimma (Ethiopia), a 168-sample size was used and included only VVF patients, unlike in this study [16].

On the other hand, the magnitude of this study was lower than the studies done in Bahir Dar (Ethiopia) (35.3%) [18], Tanzania (42.9%) [34], and Angola (58%) [7]. The possible reason might be the difference in the study period. In this study, the most recent data from 2016 to 2020 was included, unlike the previous studies of Bahir Dar (from 2013 to 2017), Tanzania (from 2014 to 2015), and Angola (from 2011 to 2016). This may emphasize the relative improvement of obstetric fistula repair failure with time, as the magnitude of obstetric fistula repair failure was shown to be lower in this study after five years. It could also be explained by differences in sample size (for example, in Tanzania, a 132-sample size was employed, whereas, in this study, a 562-sample size was used) and background characteristics of patients.

In this study, factors affecting obstetric fistula repair failure demonstrated that women who had a labor duration of more than 48 h were two times more likely to have an obstetric fistula repair failure than women who had a labor duration of less than 48 h. This finding was comparable with previous studies done in Bahir Dar (Ethiopia) [18] and Rwanda [19]. The possible explanation might be due to the presence of any type of delay, whether indecision or delay in reaching a health facility or delay in getting the quality of care, which can increase the duration of labor and lead to longer obstructed labor. This problem may result in a large degree of tissue damage, which affects fistula size and type of fistula and finally makes the fistula more complex and patients vulnerable to fistula repair failure [6].

According to the findings of this study, women with Goh Type 4 fistulas were 3.9 times more likely than women with Goh Type 1 fistulas to have obstetric fistula repair failure. This finding is supported by the findings of a study conducted in the Bahir Dar Hamlin fistula center (Ethiopia) [18]. The possible explanation might be due to the close of the fistula to the external urethral meatus or the hymen, largely affecting the function of the urinary system, which leads to high risk of fistula repair failure [35].

Additionally, the findings of this study demonstrated that women who had large fistula sizes (> 3 cm) were 6.6 times more likely to have obstetric fistula repair failure compared to women who had less than or equal to 3 cm. This finding was supported by studies done in Bahir Dar (Ethiopia) [18], the Democratic Republic of Congo [36], Uganda [14], and Pakistan [8]. The plausible explanation might be due to the large fistula being difficult to mobilize fully or there being little bladder tissue left to achieve a tension-free repair. Due to this, it leads to difficulty in closing [20, 21].

Moreover, this study revealed that those women with a total damaged urethra were 3.2 times more likely to experience obstetric fistula repair failure compared to women with an intact urethra. This finding is supported by findings from the studies conducted in Addis Ababa fistula hospital (Ethiopia) [37], Guinea [10], Democratic Republic of Congo [38], and Cameron [39]. The possible reason might be due to the urethra’s length being affected, and the damaged urethra becoming denervated and shortened. In fact, urethral fistula repair is a complex procedure, which results in patients being incontinently even after surgical repair is done [4, 34, 35].

Similarly, this study also showed that those women who had bladder catheterization for > 14 days were 2.9 times more likely to have obstetric fistula repair failure. A previous study finding of the Mekelle Hamlin fistula center (Ethiopia) supports this.[23]. The possible explanation is that the urinary bladder catheterization for more than 14 days might “increase risk of pain, infection, and erosion related to the catheter,” and this finding highlights the importance of considering WHO recommendations on the number of days for bladder catheterization for women who underwent surgical repair of fistula. The WHO recommends a short duration of bladder catheterization (7–10 days) during the postoperative period for women who have undergone surgical repair of an obstetric fistula [42].

### Limitation of the study

Important variables such as Waaldijk’s classification of the fistula and age at first marriage were not available. In addition, there were unknown data (no information) for some variables. Moreover, this study revealed that in the majority of cases, obstetric fistula repair failure was due to incontinence after successful closure of the fistula. The causes of incontinence might be different factors (for example, stress and/or urge incontinence) [37]. However, this study failed to assess the possible causes of incontinence after successful closure. Furthermore, this study assessed obstetric fistula repair failure at the time of discharge (after 21 days). However, the condition might be changed within a three-month follow-up period after obstetric fistula repair has been done.

## Conclusions

This study showed a decrease in the magnitude of obstetric fistula repair failure in a five-year period. However, the overall magnitude of obstetric fistula repair failure was high, which is above the WHO standard. Longer duration of labor, Goh Type 4 fistulas, large fistula size, total urethra damage, and longer duration of bladder catheterization were positively associated with obstetric fistula repair failure.

Therefore, Yirgalem Hamlin fistula center has better ascertain caution during surgical repair of fistula patients who have a longer duration of labor, Goh Type 4 fistulas, large fistula size, and total urethra damage. In addition, all health care providers who work in maternity units, including fistula centers, should have to educate women about the risks of a longer duration of labor. Moreover, our findings demonstrate the importance of further research on problems of incontinence after successful closure, and a prospective study is recommended to get variables not included in this study. Overall, stakeholders (government, non-government organizations, and policymakers) with the collaboration of fistula centers should give priority to implementing interventions on associated factors of obstetric fistula repair failure to reduce or prevent the failure of obstetric fistula repair.

## Supplementary Information

**Additional file 1**. Waaldijk Classification and Goh Classification of Obstetric Fistula.

## Data Availability

The datasets used and/or analysed during the current study are available from the corresponding author on reasonable request.

## Abbreviations

ANC: Antenatal Care
AOR: Adjusted odds ratio
CI: Confidence interval
COR: Crude odds ratio
HIV: Human immunodeficiency virus
RVF: Recto vaginal fistula
SD: Standard deviation
SPSS: Statistical Package for Social Science
VIF: Variance inflation factor
VVF: Vesicovaginal fistula
WHO: World Health Organization
