# Supplementary material for: Obstetric fistula repair failure and its associated factors among women underwent repair in Yirgalem Hamlin fistula center, Sidama Regional State, Southern Ethiopia, 2021: a retrospective cross sectional study
Source: BMC Womens Health. 2022 Jul 10;22:288. doi: 10.1186/s12905-022-01866-z (PMC9272558; doi:10.1186/s12905-022-01866-z)
Supplement: Supplementary file 1 — Additional file 1. Waaldijk Classification and Goh Classification of Obstetric Fistula. [file 12905_2022_1866_MOESM1_ESM.doc]

Additional file 1

## Waaldijk Classification and Goh Classification of Obstetric Fistula

| Classification of fistula based on two systems | |
| --- | --- |
| Waaldijk classification | |
| Type I | Not involving the urethral closing mechanism |
| Type II | Involving the urethral closing mechanism  A: Not involving (sub) total urethra:  Aa: without circumferential defect and  Ab: with circumferential defect  B: Involving (sub) total urethra (Urethral length < 1.5 cm):  Ba: without circumferential defect and  Bb: with circumferential defect |
| Type III | Miscellaneous, e.g. ureterovaginal fistula |
| **Goh Classification** | |
| Site | Based on the distance of the distal edge of fistula from the external urethral orifice (EUO) for VVF and/or hymen for RVF :  Type 1: > 3.5 cm,  Type 2: 2.5 -3.5 cm,  Type 3: 1.5- < 2.5 cm,  Type 4: < 1.5 cm |
| Size | a. < 1.5 cm in the largest diameter  b. 1.5 – 3 cm in the largest diameter  c. > 3 cm in the largest diameter |
| Scarring | i. None or only mild fibrosis and/or vaginal length > 6 cm normal capacity  ii. Moderate or severe fibrosis and/ or reduced vaginal length < 6cm capacity  iii. Special considerations; e.g. ureter involvement and circumferential fistula |
